# RDH12 retinopathy: clinical features, biology, genetics and future directions

**DOI:** 10.1080/13816810.2022.2062392

**Published:** 2022-05-02

**Authors:** Malena Daich Varela, Michel Michaelides

**Affiliations:** a UCL Institute of Ophthalmology, University College London, London, UK; b Moorfields Eye Hospital, London, UK

**Keywords:** *RDH12*, early onset severe retinal dystrophy, LCA, gene therapy, retinal dystrophy, molecular genetics

## Abstract

Retinol dehydrogenase 12 (RDH12) is a small gene located on chromosome 14, encoding an enzyme capable of metabolizing retinoids. It is primarily located in photoreceptor inner segments and thereby is believed to have an important role in clearing excessive retinal and other toxic aldehydes produced by light exposure. Clinical features: RDH12-associated retinopathy has wide phenotypic variability; including early-onset severe retinal dystrophy/Leber Congenital Amaurosis (EOSRD/LCA; most frequent presentation), retinitis pigmentosa, cone-rod dystrophy, and macular dystrophy. It can be inherited in an autosomal recessive and dominant fashion. RDH12-EOSRD/LCA’s key features are early visual impairment, petal-shaped, coloboma-like macular atrophy with variegated watercolour-like pattern, peripapillary sparing, and often dense bone spicule pigmentation. Future directions: There is currently no treatment available for RDH12-retinopathy. However, extensive preclinical investigations and an ongoing prospective natural history study are preparing the necessary foundation to design and establish forthcoming clinical trials. Herein, we will concisely review pathophysiology, molecular genetics, clinical features, and discuss therapeutic approaches.

## Introduction

Retinoids are photosensitive molecules of key importance in vision and cellular differentiation (1). Retinol dehydrogenase 12 (RDH12) is one of the enzymes that metabolizes retinoids within photoreceptors. It belongs to the short-chain dehydrogenases/reductases superfamily and is highly expressed in photoreceptor inner segments (2,3).

Variants in *RDH12* (MIM 608830) have been associated with autosomal recessive (AR) early onset severe retinal dystrophy/Leber Congenital Amaurosis (EOSRD/LCA), cone/cone-rod dystrophy, retinitis pigmentosa (RP), and macular dystrophy (MD); and autosomal dominant (AD) RP (4). Milder phenotypes have also been recently described in individuals with an AR inheritance pattern (5,6). Biallelic variants in *RDH12* account for 3.5–10.5% of all EOSRD/LCA cases, with a higher prevalence in East Asian population (7,8).

## Role in vision

The visual cycle is the process that occurs in photoreceptors and retinal pigment epithelium (RPE), enabling perception of visual stimuli by recycling vitamin A (all-trans-retinol) (9). As this molecule is oxidized, esterified, reduced, and hydrolysed, it becomes a substrate to different enzymes such as RDHs. Among these, RDH8 and RDH12 are primarily responsible for the oxidation and reduction of all-trans-retinoids in the outer and inner segment, respectively, of rods and cones (2,10). The RDH12 enzyme has dual specificity, with both all-trans and 11-cis-retinoids being substrates (1).

**Figure 1.**
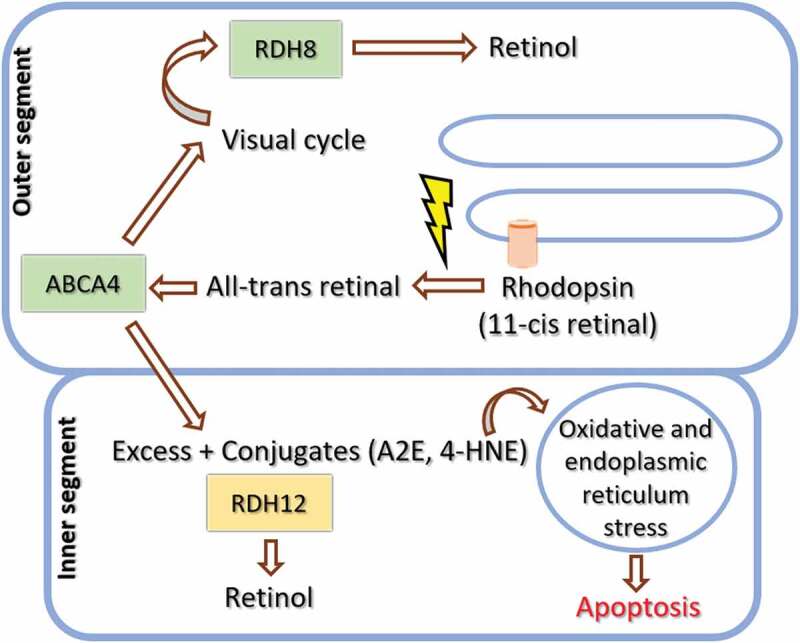
RDH12 role within photoreceptors. Once light changes the configuration of 11-cis retinal to all-trans retinal, it gets released from rhodopsin (or cone opsin) and transported from inside of the photoreceptor discs to the cytoplasm by ABCA4. RDH8 is located in this zone of the outer segment and reduces most all-trans retinal to all-trans-retinol, within the visual/retinoid cycle. Excessive all-trans retinal and conjugation products such as A2E and 4-HNE, produced by light exposure-mediated lipid peroxidation, migrate to the inner segment and become the substrate of RDH12. The accumulation of the latter, due a poorly functional RDH12, lead to increased oxidative and endoplasmic reticulum stress, enhanced cellular sensitivity to light-induced oxidative injury, and ultimately, apoptosis.

*RDH12* is usually depicted within the visual cycle loop, at the step where all-trans retinal becomes all-trans retinol (11). However, some discrepancies have appeared when elucidating RDH12 function. In vitro work has shown that the step in which RDH12 appears to be most efficient is in the reduction of all-trans and 11-cis-retinal, in the recovery phase of the visual cycle (12–15). It has been estimated that 98% of the all-trans-RDH activity is undertaken by RDH8 and RDH12 together, 70% by RDH8 and 30% by RDH12 (16). Given that the reduction of all-trans retinal takes place primarily in photoreceptor outer segments and RDH12 is located in the inner segment, it has been postulated that its contribution in the visual cycle may be indirect or auxiliary (17). The potential roles include to reduce excessive all-trans retinal (18), A2E, and/or other aldehydes produced by light exposure-mediated lipid peroxidation, such as 4-HNE (Figure 1) (19). The accumulation of the latter is involved in stress signaling, free radical reactions, and in the activation of the apoptotic response (20). Maeda *et al*. also suggested that RDH12 may regulate the flow of retinoids in the eye, playing an important part against light-induced photoreceptor apoptosis during persistent illumination (21). Recently, RDH12 metabolizing all-trans retinal has likewise been found to be key in protecting cells from oxidative and endoplasmic reticulum (ER) stress (22).

The phenotype of double knockout mouse models (Rdh8^−/−^ Rdh12^−/−^) is mild, both histologically and with respect to retinoid homeostasis dysregulation, unlike other visual cycle enzyme animal models such as RPE65 or LRAT (3,21,23,24). Photoreceptors of Rdh12^−/−^ mice were noted to have sufficient amounts of 11-cis retinal, yet they were more prone to light-induced apoptosis than those of wild-type mice (21). This suggests that pathogenesis may indeed be due to increased cellular sensitivity to light-induced oxidative injury and the accumulation of toxic by-products, rather than a disruption in the cycling of vitamin A (16,17).

## Genetics

*RDH12* was the third recessive EOSRD/LCA gene (LCA3) to be characterized, described by Stockton in 1998 (25). It contains seven exons, spans approximately 13 kb, and encodes a 316-amino acid, 35 kD protein. RDH12 protein contains a cofactor binding site, a catalytic domain, and an amino terminal motif consisting of beta-strands and alpha-helixes (26). Little is known about RDH12 tertiary structure. Thompson *et al*. created an approximate model that depicts a globular form (15). It can interact both with nicotinamide adenine dinucleotide (NADH) to oxidise retinol to retinal, and -mainly- with nicotinamide adenine dinucleotide phosphate (NADPH) to reduce retinal to retinol (10).

At present, ClinVar shows 39 pathogenic, 32 likely pathogenic and 45 variants of unknown significance in *RDH12* (total 116, https://www.ncbi.nlm.nih.gov/clinvar, accessed December 2021). Of these, 79 (68%) were missense, 14 (12%) nonsense, nine (8%) frameshift, seven (6%) splice-site, and seven (6%) in untranslated regions (UTR). Ninety-nine were single-nucleotide changes and 17 copy-number variations such as insertions, deletions and duplications. The most frequently reported homozygous genotypes, in order of frequency, were p.(T49M), p.(A126V), p.(Y226C), p.(C201R), p.(L274P), p.(S203R), and p.(L99I) (27). p.(V146D) was the most common variant in a Chinese cohort (8), p.(C201R) in patients of Indian descent, and p.(A269AfsX1) in white British patients (28). The carrier frequency of p.(A126V) among the Israeli population was found to be 0.62% (29). Of note, p.(T49M) and p.(L99I) have been associated with milder phenotypes (27).

Most carriers of null alleles are disease-free, which means that half the concentration of RDH12 protein is still well tolerated by the retina. Certain heterozygous variants, however, have been associated with a gain of function disease mechanism and a mild RP phenotype, inherited in an AD fashion (30). These variants are c.763delG, c.778delG and c.759delC, to date, all affecting the reading frame-specific C-terminal peptide (30–32). It is likely that these frameshift variants within this particular region have a toxic effect that leads to photoreceptor death, such as was hypothesized for *RGR*-retinopathy (33). This phenomenon of a milder AD phenotype in an AR EOSRD/LCA gene has also been observed in *GUCY2D* and *RPE65* (34–36).

## Clinical phenotypes

**Figure 2.**
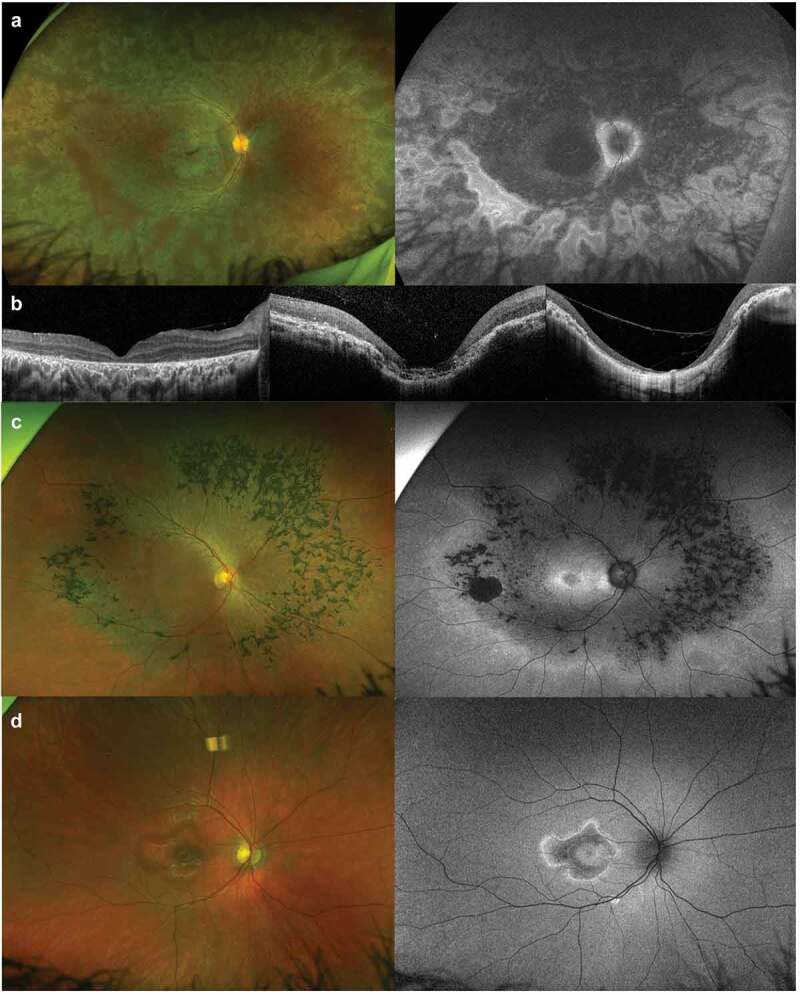
*RDH12*-retinopathy fundus features. A) Ultrawide colour and autofluorescence fundus imaging of a 12-year-old boy with early onset severe retinal dystrophy (EOSRD). The macular atrophy presents the characteristic variegated watercolour-like pattern extending to the periphery. Peripheral retinal pigment epithelium (RPE) atrophy with minimal pigmented deposits, and peripapillary sparing, are also present. B) Macular Optical Coherence Tomography (OCT) from 8-, 18-, and 39-year-old individuals with EOSRD. We see loss of the outer layers with preserved overall retinal structure in the youngest patient, and a progressive loss of retinal architecture along with coloboma-like lesion formation in the older patients. C) Ultrawide fundus imaging from a 35-year-old individual with autosomal dominant retinitis pigmentosa. The mid-periphery appears mostly involved with pigmented bone spicules and RPE loss, while the macula and far periphery remains preserved. D) Ultrawide fundus imaging from a 13-year-old patient with macular dystrophy. We see a perifoveal area of atrophy with a hyperautofluorescent rim, preserved central and peripheral structure.

*RDH12*-EOSRD/LCA can present with certain phenotypic features that aid the clinical diagnosis. Macular atrophy is common, appearing also as disorganised retinal layers on optical coherence tomography (OCT) (28,37). These atrophic changes may look like yellowish discoloration early on, followed by confluent pigment deposits, and finally a petal-shaped, coloboma-like configuration (Figure 2a a nd b) (8). The macular atrophy has also been described as having gold foil-like reflectance and a variegated watercolour-like pattern, which sometimes extends to the periphery and is readily identified by fundus autofluorescence (FAF) imaging (7,38). Progressive macular degeneration usually starts in early childhood and, although it is not pathognomonic for *RDH12* and can also be seen in *CRB1-, NMNAT1-* and *AIPL1-*EOSRD/LCA, it certainly helps to refine the possible genotypes (27,39–41). Other common features are early peripheral RPE atrophy with pigmented deposits, including bone spicules, appearing in late childhood or early adulthood (42), and peripapillary sparing (best seen with FAF) (43). Functionally, it is a severe dystrophy with markedly reduced scotopic and photopic electroretinogram (ERG) responses as early as 1 year of age, visual impairment in infancy/early childhood, and usually legal blindness before the third decade of life (7,8,42,44).

*RDH12*-RP has been reported to cause symptoms from the second or third decade, with maintained visual acuity, attenuated rod more than cone ERG responses, and driving capabilities until late adulthood (32). The mid-periphery is the most affected area and, in contrast to the EOSRD/LCA presentation, macular structure tends to remain preserved (Figure 2c) (31). MD secondary to *RDH12* biallelic variants can present as a fovea-sparing maculopathy with normal/mild-moderately reduced cone ERGs and normal rod function (Figure 2d) (45); while *RDH12*-CORD usually presents with broader compromise of the posterior pole, spreading beyond the arcades, with peripapillary sparing in younger patients (5,8). Onset of visual disturbance is variable but can be as late as in the 30s (depending on foveal involvement), with progressive loss of central and peripheral vision over time (5).

## Therapeutic options

The inherited retinal dystrophy field has been in the spotlight due to the expansion of gene therapy approaches for multiple targets, with the approval of Luxturna being the proof of a successful gene supplementation approach in *RPE65*-EOSRD/LCA (46,47). *RDH12* is attractive therapeutically for several reasons, including its small size. One of the main challenges has been the lack of informative animal models, as discussed earlier. However, the recent development of alternative assays/models such as an in vitro human cell line expressing mutant RDH12, an in vivo mutant zebrafish model (22), and induced pluripotent stem cell-derived retinal models from patients with *RDH12*-retinopathy, provide promising platforms for further understanding the biology and delineating treatments (48).

Feathers *et al*. have developed a recombinant adeno-associated viral (rAAV) vector that packed the entire *RDH12* coding region, which they tested in Rdh12^−/−^ mice (49). After a 1-year follow-up, they did not find evidence of retinal damage or disturbances in retinoid metabolism, suggesting that rAAV2/5-hGRK1p.hRDH12 could be a therapeutic candidate. Bian *et al*. also recently published a model in which they induced retinal degeneration in Rdh12^−/−^ mice by exposing them to bright light, and reported a delay in photoreceptor degeneration in mice treated with AAV2/8-mRdh12 (50). Thus, preclinical data on gene supplementation has shown promising results.

Antioxidants, retinal scavengers, and ER-stress lowering drugs have also been investigated as potentially less invasive approaches (22,51). Pregabalin, an FDA-approved drug for nerve pain, anxiety, and epilepsy treatment (52), was found to protect the retina from light-induced damage in Rdh12^−/−^ mice and *RDH12* mutant cell lines, capturing free all-trans retinal and decreasing its conjugation products and ER stress markers (51). These types of approaches are also under investigation to treat Stargardt disease, caused by variants in another visual cycle gene, *ABCA4*. Its pathophysiology also entails the build-up of retinoids and its fusion products within the photoreceptors disc membranes/RPE (53). Treatments to decrease the formation of retinaldehyde (visual cycle modulator -emiustat-, NCT03772665; deuterated vitamin A -ALK-001-, NCT02230228), inhibit the inflammatory complement cascade (avacincaptad pegol, NCT03364153), and improve antioxidant activity (omega-3 fatty acids, NCT03297515; saffron, NCT01278277) are currently in clinical trial phase (54). If successful, these may also be effective for *RDH12*-retinopathy.

Regarding clinical research, necessary, prospective, natural history studies need to be undertaken in order to determine suitable outcome measures, characterize the disease rate of progression, and define a window of opportunity for intervention. A multicentre prospective natural history study is on-going, recruiting both children and adults (USA and London, UK). This will lay the groundwork for future planned interventional studies.

In summary, there is on-going evaluation about RDH12ʹs role(s) in vision and how when aberrant it causes disease; currently believed to be primarily related to defective clearance of toxic by-products and/or oxidative and ER stress. *RDH12*-retinopathy can be inherited both in AR and AD patterns and can be associated with wide-ranging severity, with EOSRD/LCA being the most frequently reported condition (44). *RDH12*-EOSRD/LCA is characterized by early macular atrophy and often legal blindness before the third decade. The increasing knowledge about its molecular basis, the promising preclinical data on gene supplementation, and the ongoing natural history study, raise cautious optimism for patients and families.
